# Read. This. Slowly: mimicking spoken pauses in text messages

**DOI:** 10.3389/fpsyg.2025.1410698

**Published:** 2025-02-10

**Authors:** Rachel C. Poirier, Andrew M. Cook, Celia M. Klin

**Affiliations:** Department of Psychology, Binghamton University, Binghamton, NY, United States

**Keywords:** computer-mediated communication, CMC, texting, text messaging, language processing, language comprehension

## Abstract

In contrast with face-to-face conversations, text messages lack important extralinguistic cues such as tone of voice and gestures. We ask how texters are able to communicate the same nuanced social and emotional meaning without access to this rich set of multimodal cues. The current paper expands on previous work examining the role of one particular textism, the period, and found that the inclusion of a period after a single-word text (yup.) could convey abruptness, or insincerity. Across three experiments, we used a rating scale to examine two additional textisms and found that the inclusion of a period after each word in an exchange (No. Just. Go) as well as breaking the exchange into a series of single-word texts ([no] [just] [go]) conveyed emotions such as disgust and frustration. These textisms may have mimicked prosody, influencing readers’ understanding of the emotionality of the message. More generally, the results demonstrate that texters make use of a variety of textisms to communicate social and emotional information.

## Introduction

Digital forms of written communication, such as texting, serve a communicative role that is similar to spoken language. Texting, or “talk writing” (McWhorter, 2012), allows for a rapid exchange between people, often in an informal, conversational style. In this way, texting provides an interesting linguistic challenge: Spoken conversations rely heavily on cues that are not available during texting --linguistic elements such as prosody, which consist of the patterns of intonation, stress, and rhythm that shape meaning, as well as nonverbal elements, such as eye contact, facial expressions, and gestures (e.g., Banzinger and Scherer, 2005; Knapp et al., 2013). All of these cues, prosodic and nonverbal, can convey important social and pragmatic information that can significantly influence meaning. We ask if, and how, texters are able to communicate the type of nuanced meaning that is present in face-to-face conversations without the same set of multimodal cues.

Early research suggests that some of the multimodal cues used in a spoken conversation are replaced by *textisms*, digital signals such as emoticons and emojis, abbreviations, intentional misspellings, and nonstandard punctuation. These types of cues may serve to disambiguate a message, mimic the phonology of speech (e.g., gonna; Altohami, 2020), add emotion (Riordan and Kreuz, 2010; Upadhyay et al., 2023), or fundamentally change the meaning of a message, allowing texters to convey the range of nuanced meanings available during spoken conversations (Kulish, 2021).

Many of these findings came from research that analyzed naturally occurring texts in non-experimental designs, which conjectured about the meaning senders hoped to convey through the inclusion of various textual cues (e.g., Altohami, 2020; Herring et al., 2013). Multiple corpus analyses have analyzed patterns in naturally occurring texts (e.g., Asprey and Tagg, 2019; Kulish, 2021; Rodríguez-Hidalgo et al., 2017; Wright, 2018). More recently, empirical studies have examined receivers’ understanding of a range of textual devices rather than senders’ intended meanings. For example, Filik et al. (2016) asked participants to read short texts and found that emoticons added to the emotional impact of sarcastic messages. In Riordan (2017), participants read positively and negatively valenced texts; generally, emoticons conveyed positive affect. Wright (2018) had participants rate tweets with varied punctuation along eight emotional dimensions and found that emotional ratings depended on both the type of punctuation and the linguistic context. In Reynolds et al. (2017), participants read text messages that ended with a range of punctuation marks. Punctuation was interpreted pragmatically, with different punctuation marks influencing the participants’ text responses and their understanding of the of the sincerity of the message. In other words, the punctuation conveyed a conversational tone.

In a series of experiments, Gunraj et al. (2016) and Houghton et al. (2018) examined the meaning of one specific type of textism: the period, or full stop. They hypothesized that punctuation in text messages might be used in a way punctuation was used in antiquity, when written language was a guide to reading aloud. Punctuation told the reader where to pause, which words to emphasize, and where to take a breath. Although the role of punctuation in written language has evolved through the centuries to be increasingly grammatical rather than rhetorical, or pragmatic, providing information about the structure of the sentence rather than its meaning (Baron and Ling, 2011), digital communication has been referred to as *talk-writing* (McWhorter, 2012) or *digitalk* (Turner, 2010), and thus, punctuation could again be conveying intonation and prosody in this medium.

To examine these ideas, Gunraj et al. (2016) and Houghton et al. (2018) asked participants to evaluate one-word text messages that either included a period (yup.) or did not (yup). Overall, a period led readers to understand the message as less sincere or more abrupt. This effect was not found when the materials were handwritten notes instead of text messages, suggesting, minimally, that punctuation is understood differently in digital communication than in other forms of communication. Houghton et al. concluded that although periods are not inherently negatively charged (e.g., Albritton, 2022), adding a period to a single-word text exchange conveyed the intent to communicate abruptness or finality.

Houghton et al. (2018) hypothesized that because the texts they examined consisted of single-word, casual, exchanges, readers did not interpret the period as being used grammatically. First, a one-word text message does not need a period to indicate that the communication is complete. The message-final periods examined by Houghton et al. are in contrast with messages containing message-medial periods (e.g., “Thanks for dinner. I’ll be…”), which serve a grammatical, rather than pragmatic, function and are understood as they would be in formal written language—to indicate the end of a unit of meaning (e.g., Albritton, 2022). Consistent with this, periods are often left out at the end of text messages: “(T)he act of sending a message coincides with sentence-final punctuation” (Baron and Ling, 2011). Second, it has been argued that the more informal the text exchange, the more likely the exchange will mimic spoken structures. Thus, it is unsurprising that the grammatical principle of punctuation, in which punctuation is used to mark syntactical structure, is sometimes replaced in digital communication by a rhetorical principle, in which punctuation facilitates a shared meaning between the conversational partners, perhaps providing an intonational structure (Busch, 2021).

How was the period understood after a one-word text (e.g., yeah.)? According to Grice’s conversational maxim of relevance (Grice, 1975), people expect their conversational partner’s contributions to be relevant and meaningful. Given this, readers assumed that the inclusion of a period was deliberate, relevant, and meaningful. Because it was not needed to convey grammatical information, it was assumed to convey pragmatic information. More specifically, readers understood the period as communicating finality and abruptness, perhaps mimicking the vocal prosody of a dramatic pause.

Consistent with Grice’s maxim of relevance, Riordan (2017) posited that participants interpret object emojis as conveying positive affect due to the sociological principle of “emotion work;” that is, text recipients understood that the inclusion of an emoji required effort, thus conveying emotional outreach. Similarly, the effort involved in including a message-final period, when it was not needed grammatically, may communicate to the text recipient that an effort was made to add meaning, perhaps intonation that is akin to a pause in vocal prosody. In turn, the period adds negatively or harshness (Albritton, 2022). “To younger people, putting a period at the end of a casually written thought could mean that you are raring for a fight” (Harrison-Caldwell, 2021). Kulish (2021) refers to a period in a text message as the “dot of hate.” Based on the unique meaning a period can carry in a text, Adams and Miles (2023) have argued that texters respond by employing the *markers-missing* textism — the lack of a period in order to avoid communicating this type of negativity.

The current set of experiments furthered the investigation of how texters communicate nuanced meaning without the rich set of cues available in spoken language. We examined two new text constructions: text messages in which a period follows each word and text messages that are broken into a series of one-word messages. Both the nonstandard use of the period and the unusual visual presentation serve as *markers-extra* (Adams and Miles, 2023). We ask if these communicate intensity or emotion, being interpreted pragmatically by readers. To examine this question, we used a design similar to that of Gunraj et al. (2016) and Houghton et al. (2018) in which participants read text messages with or without the markers-extra, and were asked to assess their understanding of the message writer’s emotional tone, rating an emotion such as disgust or anger. Although not an online measure such as eye movements, this dependent measure required little reflection on the part of the reader and a relatively fast response. Further, this provided a quantitative comparison between two conditions that varied only in the presence or absence of the critical stylistic variable.

## Experiment 1

Like encountering a period after a one-word text, periods after each word in a text message serve no grammatical function. However, given readers’ assumption that writers are deliberate in their choices, the additional periods should be understood as relevant and meaningful (Grice, 1975), conveying pragmatic information such as emotion. Adams and Miles (2023) refer to textisms that involve nonstandard punctuation as *markers-extra*. Markers-extra are, not surprisingly, used to communicate an increased sense of intensity or emotion:

As a blogger for a viral content website, I can tell you there’s just one thing that bloggers like me are after, day in and day out: crafting that One. Perfect. Sentence. That. Is. Both. Evocative. And. Concise. And while we often use one simple trick to drive a point home, what I found out next shocked me: I’ve been putting a period after Every. Single. Word. Way. Too. Early. In. My. Sentences. Thus. All. But. Negating. The. Intended. Dramatic. Effect. (Blair, 2016).

We hypothesize that adding a period communicates drama and amplifies negative emotions such as anger, frustration, or disgust. We expect that people will read texts with markers-extra, specifically with periods after each word, as more emotional than texts sent without such markers.

### Methods

#### Participants

Participants were 80 undergraduate students from Binghamton University. They received credit toward a course requirement in exchange for their participation. Data were collected online. Participants only participated in one of the experiments reported in this paper. All experiments were conducted following the guidelines of the Binghamton University institutional review board.

#### Materials

A series of text messaging exchanges were created using an iPhone Text Generator.^1^ All text messages were exchanges between two people. A unique name appeared at the top of each screen, which, based on texting conventions, belonged to the texter on the left side of the screen. Participants were shown an example text with name labels in the instructions to familiarize them with the phone conventions. See the Appendix A. A maximum of eight texts were sent and received in each text conversation.

**Figure 1:**
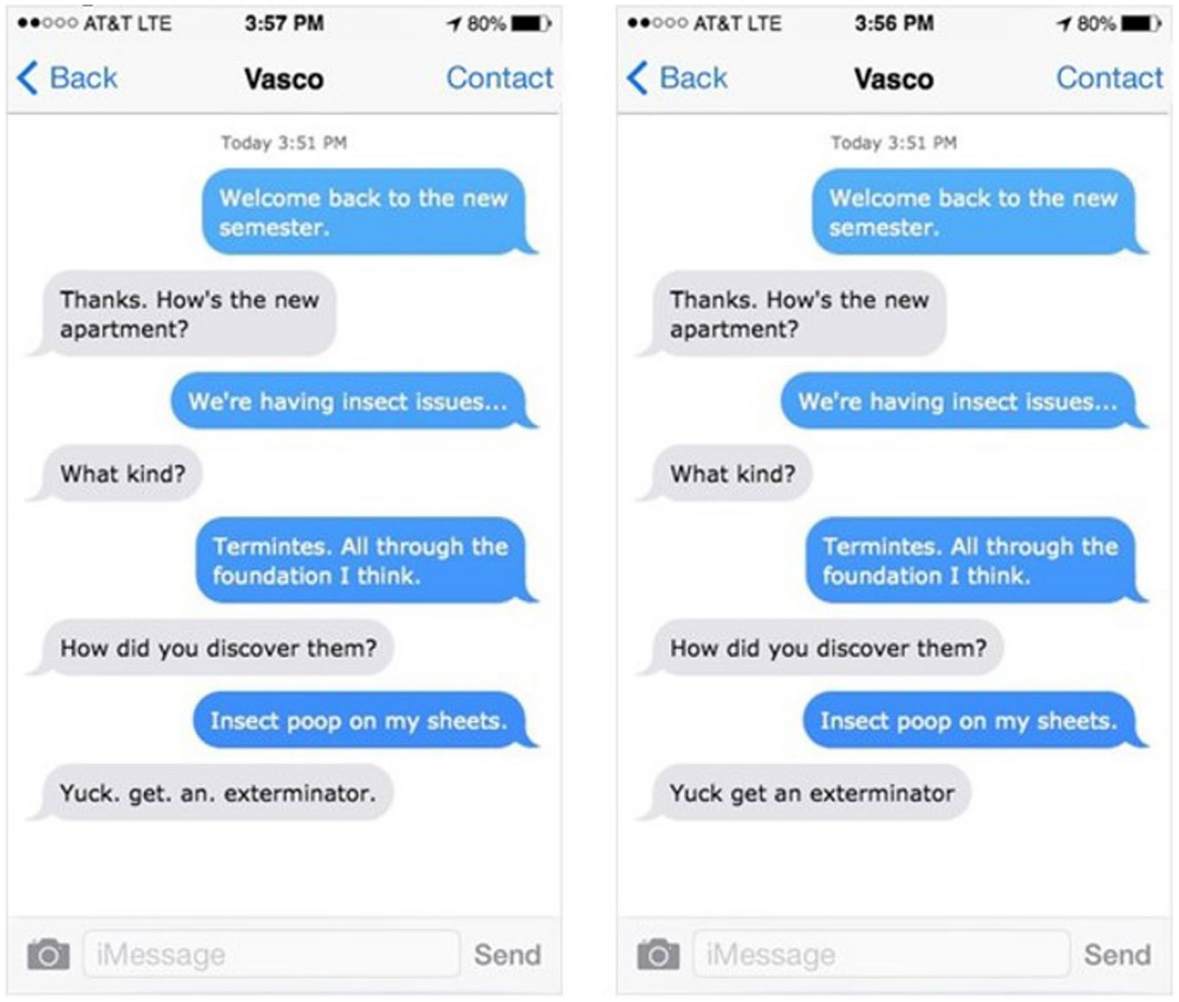
Experiment 1. Sample texts-Period and No Period versions.

The experimental items in a Period condition included a critical text in which each word in the message was punctuated by a period. See Figure 1. These critical texts conveyed negative emotions—frustration or disgust. Alternatively, in the No Period condition, the same message was sent but without the periods. There were 10 items for each emotion, resulting in 20 critical items. In a between-subjects design, participants read half of the critical items in the Period condition and half in the No Period condition. Items were counterbalanced across participants. Critical items ranged from 3 to 4 words. The critical texts were interspersed with 37 filler texts, which included a variety of types of emotional content and punctuation, and were included to obscure the manipulation. Participants rated the emotion (frustration or disgust) of each text exchange on a seven-point Likert scale. The seven-point scale was consistent with previous literature that explored periods in text (e.g., Houghton et al., 2018), but utilized different anchors (1 = Not at all, 4 = Moderately, 7 = Extremely) to approximate a continuous measurement (Casper et al., 2020).

#### Procedure

Participants signed up for the experiment through SONA, an online experimental participation platform, and received a link to the experiment, conducted on the Qualtrics platform. After providing informed consent, participants were instructed to remove all distractions. They performed the experiment on their own personal computers. Participants were instructed to put away any distractions and focus on the computer screen. They read the instructions (see Appendix A) and then proceeded through the experimental and filler text exchanges, one at a time.

For each text exchange, participants rated how disgusted or frustrated they believed the texter was (e.g., “How frustrated was Vasco in this text exchange?”). Ratings were made on a Likert scale from 1 (*Not at all*) to 7 (*Extremely*). The mid-point of the Likert scale was anchored with the slider on 4 (*Moderately*); participants had to click on the slider to make their response even if they selected the moderate option. The emotion conveyed in the text (e.g., frustration) never appeared in two consecutive trials. Further, filler texts were interspersed and pseudorandomized among the critical texts so that there were never two critical texts in a row. Five filler texts preceded the first critical text.

After completing the experiment, participants were asked if they had multitasked during the experiment due to the lack of oversight of participants in online studies: *Please be honest (this will not affect your participation credit in any way), did you multitask at any point during this experiment?* Eleven participants reported multitasking during the experiment but their data did not differ from participants who did not multitask (*p* > 0.25); therefore, multitaskers’ data were not excluded from the analysis. The experiment took approximately 30 min.

#### Results and discussion

**Figure 2:**
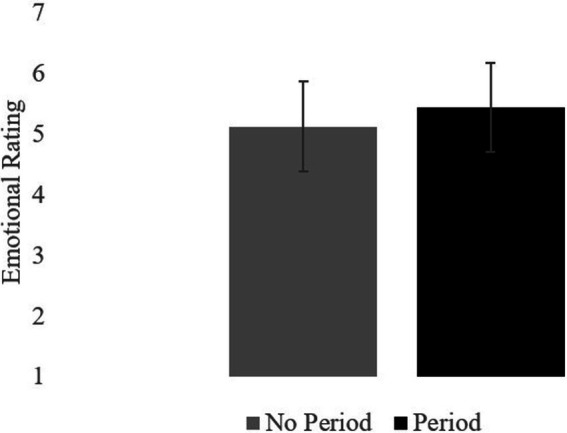
Experiment 1. Mean ratings.

All statistics were conducted using JASP 0.16.1 software. Mean ratings for the two conditions are summarized in the table. Participants rated texts in the Period condition as more emotionally intense (*M* = 5.45, *SD* = 0.74) than texts in the No Period condition (*M* = 5.13, *SD* = 0.74); *t_1_*(79) = 4.65, *SEM* = 0.08, *p* < 0.001. *t_2_*(19) = 5.18, *SEM* = 0.14, *p* < 0.001. This difference constituted a very large effect size, *d* = 1.16. See Figure 2. Readers interpreted the period after each word as conveying emotional intensity. Although the experiment was not designed to compare the two emotions, the pattern was the same for disgust and frustration.

The results are consistent with earlier findings that periods in text messages can act as nonverbal cues, communicating important social and pragmatic information such as emotion. Including a period after each word is deliberate on the part of the person writing the text message and, given Grice’s maxim of relevance, and Riordan’s (2017) ideas about *emotion work*, readers interpreted the periods as having been included in a way that was intentional and meaningful. The periods may communicate emphasis or may mimic a prosodic feature such as a dramatic pause after each word, being heard as staccato speech in readers’ inner speech. Regardless of the exact mechanism, the periods influenced readers’ understanding of the emotionality of the message.

## Experiment 2

Why did the construction in the Period condition of Experiment 1 so strongly convey emotion? Part of the answer is that including a period after each word has no grammatical function and thus, was understood rhetorically to add emphasis and convey emotion. Further, this construction might be a textism that has developed this particular meaning over time. There are certainly sets of textisms that have been implicitly agreed upon by language communities (e.g., Adams and Miles, 2023). The inclusion of a period after each word may be one of those.

In Experiment 2, we contrast the manipulation in Experiment 1 – a period after each word – with a textism that is similar in construction but may be less frequent. Instead of placing a period after each word, the critical phrases were sent as a series of individual words, with each text message containing a single word. We ask if this construction, like the Period version of Experiment 1, will communicate the same meaning -- negative emotions such as frustration, disgust, or anger. Texts again appeared in two versions. However, instead of a Period version, with a period appearing after each word of the critical phrase, we examined a Multi-text version, in which the phrase was broken into a series of single-word texts.

### Methods

#### Participants

Participants were 60 undergraduate students from Binghamton University. They received credit toward a course requirement in exchange for their participation. Data were collected online. Data from five participants were excluded because they did not complete the experiment. All participants were native English speakers.

#### Materials

**Figure 3:**
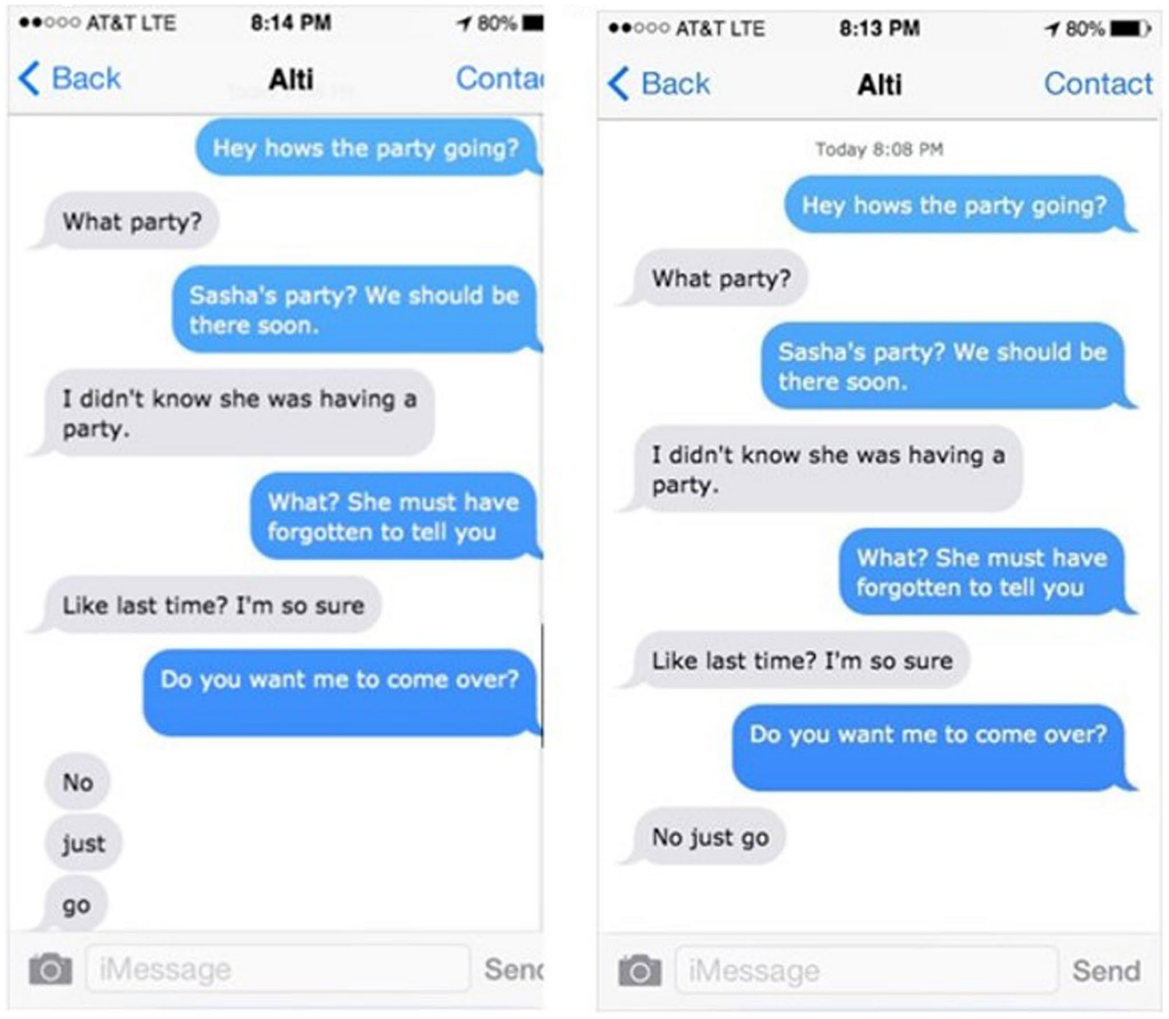
Experiment 2. Sample texts-multi text and single-text versions.

Text messages were created using an iPhone Text Generator (see footnote 1). Two conditions were created: Multi-text and Single-text. In the Multi-text version, each word of the message appeared in an individual text message (see Figure 3). This condition was similar to the Period version in Experiment 1, except the words were separated into individual text messages instead of being separated by periods. The Single-text version mimicked the No Period version in Experiment 1: Three- or four-word messages were presented in a single text message without punctuation. There were six items for each emotion - disgust, frustration, and anger, resulting in 18 critical items. Participants read each text in only one of the two versions, in a between-subjects design. Thirty-three filler texts were also included and contained a range of emotions and types of punctuation.

#### Procedure

The procedure was identical to Experiment 1. Eleven participants indicated they multitasked during the experiment; their data did not differ significantly from that of the participants who did not report multitasking (*p* > 0.5), so their data were included.

#### Results and discussion

**Figure 4:**
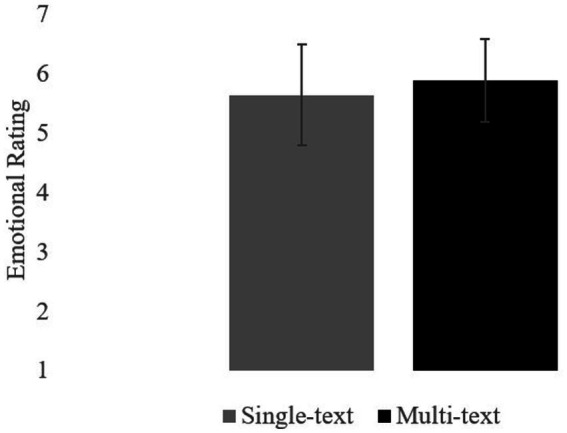
Experiment 2. Mean ratings.

Mean ratings are summarized in the table. Overall, participants rated the texts in the Multi-text version as more emotional (*M* = 5.89, *SD* = 0.70) than the texts in the Single-text version (*M* = 5.65, *SD* = 0.85); *t_1_* (59) = 2.82, *SEM* = 0.09, *p* < 0.05, *d* = 0.36; *t_2_* (17) = 2.13, *SEM* = 0.11, p < 0.05, *d* = 0.50. See Figure 4. The pattern was the same as in Experiment 1, although the effect sizes were considerably smaller.

Breaking a sentence into a series of one-word text messages changed readers’ understanding of the emotionality of the message. Although the construction is not commonly used in text messages, readers understood this textism as communicating a negative emotion. One explanation is that this was understood by readers as being equivalent to a dramatic pause after each word. Alternatively, the textism may have communicated a different aspect of prosody such as pitch, timbre, speech rate, or loudness, all of which are used to convey emotion (Larrouy-Maestri et al., 2024). Finally, the text may have simply been more salient. Regardless of the exact explanation, the texter’s decision to write in this non-standard way was deliberate, and the format served as *markers-extra* (Adams and Miles, 2023), and communicated an increased sense of emotion.

It is notable that the effect size was smaller than in Experiment 1. Perhaps the periods used in Experiment 1 were more visually salient, and thus, conveyed a stronger meaning. Another possibility is that the period carries as part of its meaning the direction to pause. And, finally, the inclusion of a period after each word is a frequent textism and may have an agreed upon meaning.

## Experiment 3

A text message read as a series of single-word texts added emotionality to the message and may serve a similar rhetorical function as punctuating each word with a period. In the current experiment, we explore another explanation: Readers rated the Multi-text messages as more emotional simply because the texts took up more physical space than the Single-line texts. In Experiment 3, we ask if the unusual visual layout in the Multi-text version, which increased the amount of physical space, adds to the emotional intensity of the messages.

**Figure 5:**
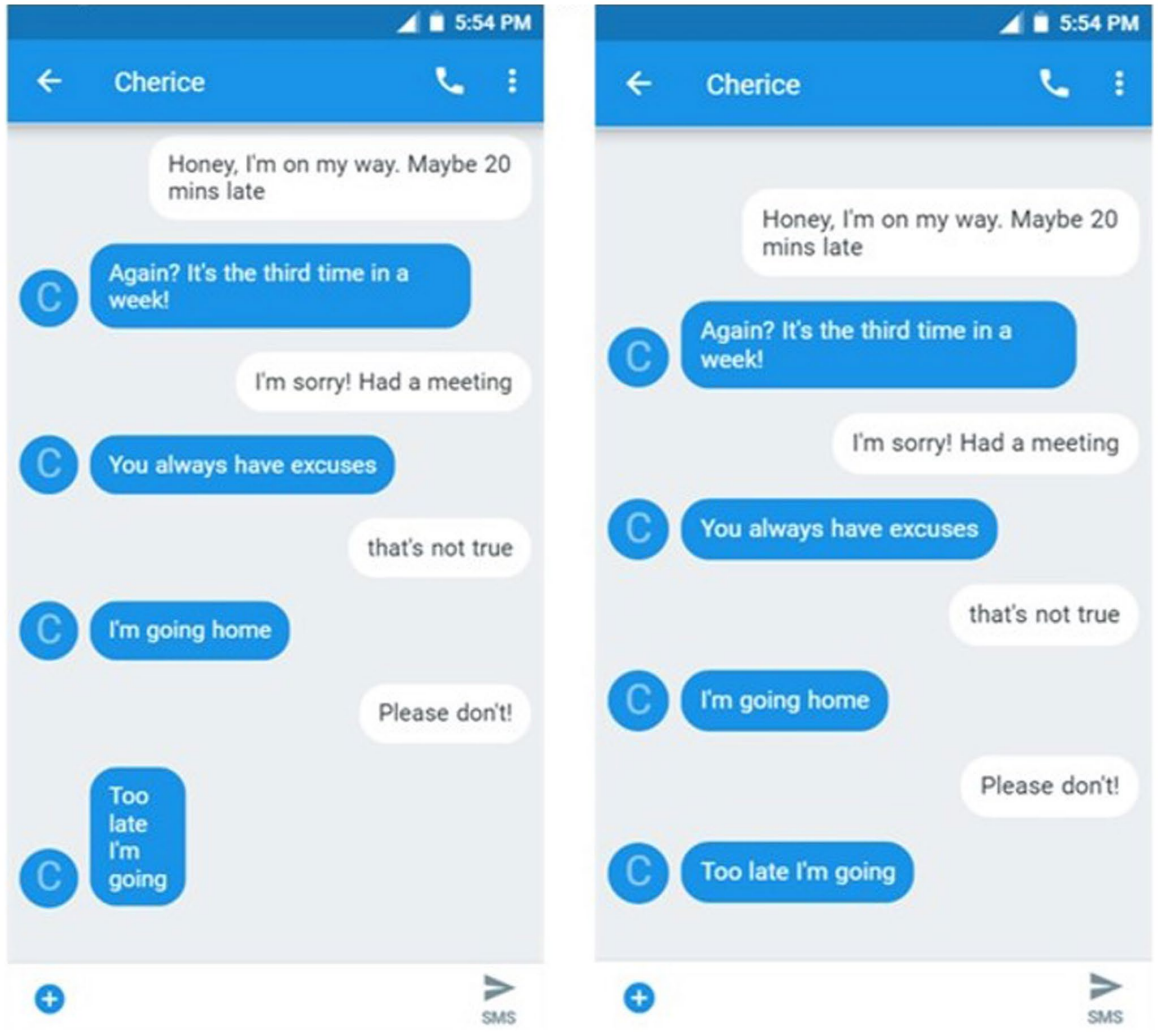
Experiment 3. Sample texts-multi line and single-line versions.

In Experiment 3, the Multi-text version is replaced with a Multi-line version. In the Multi-line version, the critical phrase was again divided into a series of individual words, with each word on one line. However, unlike the Multi-text version, all the lines appeared within the same text message. See Figure 5. The Multi-line texts were designed to act as a visual control to determine whether the increased emotionality of the Multi-text messages of Experiment 2 was due to the meaning implied by this format or to the texts taking up more visual space on the screen. The Multi-line version in the current experiment was compared to a Single-line version, the same baseline condition as was used in Experiments 1 (No Period) and 2 (Single-Text), with all of the words of the critical text on a single line and without punctuation. The question is if formatting a text message so that one word appears on each line, expanding the physical size of the text, will communicate emotionality even though the critical text is sent as a single text message.

### Methods

#### Participants

Participants were 78 undergraduate students from Binghamton University. They received credit toward a course requirement in exchange for their participation. Data were collected online.

#### Materials

The materials from the Multi-text version of Experiment 2 were modified into Multi-line messages. In the Multi-line version, the critical text was presented with each word on its own line within a single text message. See Figure 5. In the Single-line version, the critical text was presented as a single line, similar to the baseline texts in Experiments 1 and 2. Texts were re-created using an Android Text Generator.^2^ (Multi-line texts were not possible to create with the previously used generator). Each participant read 20 experimental texts—10 frustrated and 10 disgusted, as in Experiment 1— and 37 filler texts that contained a variety of emotions and punctuation marks. Participants read each text in only one version in a between-subjects design.

#### Procedure

The procedure was identical to the previous experiments. Twenty-two participants indicated that they multitasked during the experiment, but their data did not differ significantly from those who did not report multitasking (*p* > 0.3), so their data were included.

#### Results and discussion

**Figure 6:**
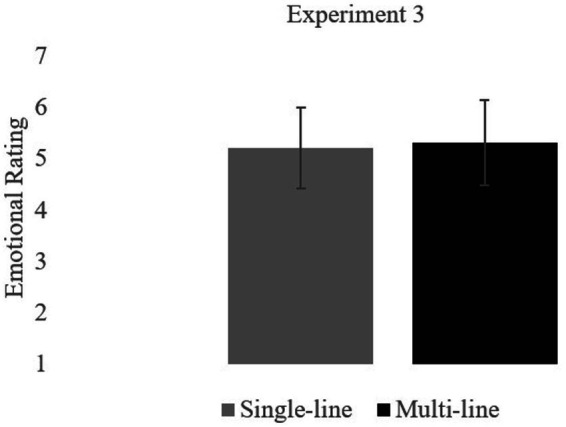
Experiment 3. Mean ratings.

Mean ratings are summarized in Table 1. The format of the text message did not influence ratings, *p* > 0.10 (see Figure 6). Whereas the text message in the Multi-line version took up more physical space and was more visually salient than the text message in the Single-line version, readers did not rate it as more emotional. This is in contrast with the Multi-text version of Experiment 2.

**Table 1:** Summary of findings—mean ratings (SD) of emotional valence.

Experiment	Versions			Difference	Effect size (*d*)
1	No Period vs. Period	5.13 (0.74)	5.45 (0.74)	0.32*	1.16
2	Single-text vs. Multi-text	5.65 (0.85)	5.89 (0.70)	0.24*	0.50
3	Single-line vs. Multi-line	5.22 (0.79)	5.32 (0.82)	0.10	*n.s.*

*p < 0.05.

The contrasting results from Experiments 2 and 3 indicate that readers do not simply perceive any unusual format as conveying meaning. Further, the emotional impact of the texts in the Multi-text version in Experiment 2 was due to something more than the visual size of the text message. Therefore, we conclude that the format of the Multi-text version, like the Period condition in Experiment 1, communicated emphasis, perhaps mimicking a spoken pause between words, which, in turn, conveyed intensity and emotion. In contrast, the format in the Multi-line version in the current experiment, with a single word per line, was read differently, perhaps more like a list. Based on the results, the format was not understood as communicating dramatic emphasis and emotion.

## General discussion

In contrast with a spoken conversation, texting does not include cues such as prosody, facial expressions, and gestures, cues that provide a great deal of pragmatic, relational, and social information. These cues are critical in spoken conversation, increasing conversational coherence, for example, by facilitating turn-taking (Darics, 2013), and allowing speakers to communicate nuanced pragmatic and social information. So, how do texters convey this type of information? Investigating this question both allows a better understanding of the communicative practices in texting and provides a window to observe the evolution of language.

In the current set of experiments, we expand on research (Gunraj et al., 2016; Houghton et al., 2018; Reynolds et al., 2017) exploring the role of punctuation in texting. In Experiment 1, we again examined the period as a textism, but with a novel construction—a period after each word in a sentence, a construction that has not been explored previously. Participants understood this construction as communicating emotion, such as disgust or frustration. We conclude, as did Gunraj et al. (2016) and Houghton et al. (2018), that periods can be used rhetorically, rather than grammatically, to alter the meaning of a phrase, perhaps by conveying prosodic information that would be found in spoken language.

In Experiment 2, we asked if other visual cues might communicate meaning. Participants rated Multi-text messages in Experiment 2 as more emotional than messages sent as a single text. Experiment 3 ruled out the explanation that it was the salience of the spatial presentation in the Multi-text condition that was critical. Messages with one word per line, but all within a single text bubble, were not rated as more emotional. Given this, the emotion conveyed in the Multi-text format in Experiment 2 was not simply due to the unusual layout or the increased screen space the messages filled. There was a specific influence of breaking a message into multiple texts, each with a single word, that may be akin to a period after each word.

The results indicate that when a period is present in a text where it is not needed grammatically (Experiment 1), or when a sentence is broken into a series of one-word texts (Experiment 2), this serves a specific communicative function. The nonstandard period and visual presentation served as *markers-extra* (Adams and Miles, 2023), communicating an increased sense of intensity or emotion. Reynolds et al. (2017) also found that punctuation could be interpreted pragmatically, influencing the meaning of a text. Although perhaps new to digital communication, the inclusion of irregularly used punctuation and spacing has a long history in fiction and poetry. For example, Ahmadjonovna, (2023) describes writers and poets who avail themselves of “author’s punctuation,” punctation that does not comply with the standard rules, as a way to add to the expressiveness of speech. Ahmadjonova assumes that the “author’s punctuation,” which is, of course, in written form, influences inner speech—the reader’s phonological representation.

The Period condition of Experiment 1 and the Multi-text condition of Experiment 2 may have been encoded by readers as a pause after each word or some other aspect of prosody. There is substantial evidence that silent reading involves phonological coding, or inner speech, “the mental representations of speech that can give rise to the experience of hearing sounds” during silent reading (Rayner et al., 2006). For example, reading a homophone (e.g., rows) activates lexical information about the other word in the homophone pair (e.g., rose). Similarly, reading similarly spelled words that are pronounced differently (e.g., nasty/hasty) slows silent reading (Treiman et al., 1983) as does reading tongue twisters (McCutchen and Perfetti, 1982). And beyond evidence about access to word-level phonology during silent reading, there is evidence that suprasegmental phonology, properties of spoken language such as stress and phrasing, influences readers’ understanding of silently-read sentences (Fodor, 1998).

The meaning of pauses in spoken language has been studied extensively, in fields from education to second language learning to AI. For example, Read et al. (2019) found that when preschoolers listened to stories in which dramatic, silent, pauses were inserted before an unfamiliar word, the children were more likely to retain that word at a delay. Corley et al. (2007) found that the filled pause, “er,” within fluent speech, helped listeners to integrate an upcoming word that was unpredictable, perhaps alerting them that the next word was going to be unpredictable, and providing them with additional attentional resources. More generally, although pauses in spoken language have been considered disfluencies, or performance errors (Chomsky, 1965), they have also been found to have specific communicative functions (e.g., Cossavella and Cevasco, 2021; Clark and Fox Tree, 2002). In this way, it is unsurprising that in “talk writing” (McWhorter, 2012), texters might find a way to communicate pauses. Busch (2021) describes digital communication as sitting on a spectrum between spoken and written language -- a “hybrid register” (Tagliamonte and Denis, 2008). Within this, he argues, punctuation plays a particularly important role in helping to create a shared understanding in interactional writing.

Whereas the current experiments demonstrate that textisms – adding a period after each word or sending texts one word at a time – influence readers’ understanding of the emotionality of the message, questions remain as to their precise influence. Although we hypothesize that a period has the prosodic quality of a pause, other possibilities exist. First, the textisms may have influenced other aspects of prosody, as emotional prosody certainly includes many features beyond the pause. For example, features such as pitch, timbre, speech rate, and loudness are all involved in communicating emotion (Larrouy-Maestri et al., 2024). Presumably, during silent reading, any of these features could be inferred and encoded into a reader’s phonological representation. And, second, the textisms may not have influenced inner speech at all, but instead, added emphasis or salience.

A limitation of the methodology used in the current experiments is that participants were asked to reflect on their understanding of the text exchanges. An online measure, such as reading times, would allow for a more direct investigation of the hypothesis that the text cues under consideration were encoded as pauses. If these textisms led readers to encode a pause after each word, reading times should be longer in the Period condition than the No Period condition, and in the Multi-Text condition than the Single-Text condition. Reading times have been successfully utilized previously to examine readers’ phonological representation during silent reading. For example, Ashby and Clifton (2005) found that words with two stressed syllables took longer to read than words with one stressed syllable in a silent reading task. It seems that the number of stressed syllables influences the time readers need to “prepare an implicit pronunciation of a word.”

Future research is also needed to investigate the range of factors that influence the comprehension of textisms. The comprehension of text messages undoubtedly depends on the communicative situation (e.g., Darics, 2013). Houghton et al. (2018) noted that their stimuli depicted casual text exchanges between friends. The same is true in the current experiments and that may be critical. Consistent with this, in evaluating text messages sent by an office in a community college, students were sensitive to the formality of the communicative situation, preferring formal writing (e.g., “you,” “to”) over textisms (e.g., “u,” “2;” Taylor and Serna, 2019). In addition, a wider range of materials could be explored. The current experiments examined texts intended to convey negative emotions, such as disgust and frustration. The period might be more likely to convey intonation in texts that convey negatively valenced emotions (Albritton, 2022) than positively valenced emotions. Reader characteristics might also influence the role of textisms in comprehension. “Digital natives,” raised when computer-mediated communication was already ubiquitous, may be more likely to interpret textisms meaningfully than “digital immigrants” (e.g., Forgays et al., 2014; Junor, 2021; Riordan et al., 2018). And finally there is variability in the richness of readers’ auditory imagery (e.g., Alexander and Nygaard, 2008). The same may be true with textisms, with variability across readers in the use and comprehension of intonational punctuation and intonational formatting.

## Data Availability

The raw data supporting the conclusions of this article will be made available by the authors, without undue reservation.

## Footnotes

^1^ http://iphonefaketext.com

^2^ https://androidfaketextmessage.com
