# Supplementary material for: Read. This. Slowly: mimicking spoken pauses in text messages
Source: Front Psychol. 2025 Feb 10;16:1410698. doi: 10.3389/fpsyg.2025.1410698 (PMC11867088; doi:10.3389/fpsyg.2025.1410698)
Supplement: Supplementary file 1 [file Data_Sheet_1.docx]

**Appendix A**

**Experiments 1, 2, and 3 Instructions**

In this task, you will see a cell phone screen on each page depicting text messages between two people. On the left side of the screen are messages in gray and on the right are messages in blue. The person sending messages in gray will have their name at the top of the cell phone screen (see example below). For each text conversation, please read the full conversation before answering the question below.
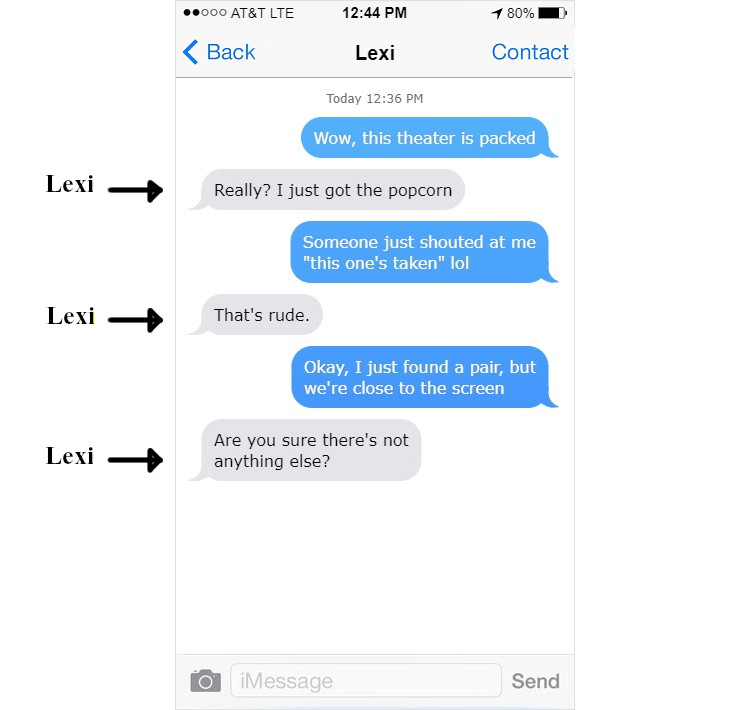


The question below the phone screen will ask you to rate an emotion for the person sending messages in gray (left side of the text conversation), whose name is located at the top of the phone screen. The rating will span 1 - 7 (1 = Not at all; 4 = Moderately; 7 = Extremely). Answer each question to the best of your ability.

When you are ready to start, scroll down and press the [arrow] to begin.
